# Hospitals with 150 Beds and Upwards

**Published:** 1916-12-16

**Authors:** 


### HOSPITAL NEEDS AND HOSPITAL APPEALS.

### Hospitals with 150 Beds and Upwards.

**BROMPTON HOSPITAL FOR CONSUMPTION AND DISEASES OF THE CHEST.**—Brompton is the leading hospital for diseases of the chest, drawing its patients from all over the country. Eleven years ago it began to send its convalescent patients out to virgin forest at Frimley, Surrey, to work themselves back to health in clearing the waste and making it a productive garden. The twofold institution—hospital and working sanatorium—is the latest scientific method of restoring consumptive sufferers to health and a useful life. Years of success have proved it ideal. In the early days of the war the resources of the Brompton Hospital were placed at the disposal of the Admiralty and War Office authorities for the treatment not only of sailors and soldiers invalided with consumption, but also of their wives and families should treatment, which this hospital with its sanatorium at Frimley is in an unrivalled position to give, be needed, and a large number of patients have received the benefit of treatment at the hospital and the sanatorium under this arrangement. Owing to the war, however, public liberality has been diverted into many other channels, and an additional annual income of £10,000 is now most urgently needed. Mr. Frederick Wood is the Secretary.

**CITY OF LONDON HOSPITAL FOR DISEASES OF THE CHEST, VICTORIA PARK, N.E.**—This hospital is amongst those labouring under heavily increased needs imposed by the war conditions. At all times hampered by its position, removed from the wealthy residential districts (admirable though it is for its work, as it adjoins the Victoria Park of 217 acres), the usual serious problem as to raising sufficient income is seriously augmented by the cost of maintenance under present conditions, adding £2,500 to its needs for the present year. It is feared that there will be a deficiency of little less than that amount on the present year's account unless substantial help is forthcoming. The value of the hospital under the present distressing conditions has increased rather than been reduced, treating as it does not only men invalided from the Forces, but also the wives and children of our sailors and soldiers. With its 175 beds and extensive out-patient department, and the municipal Tuberculosis Dispensary which is now established there, its work is of immense importance. Contributions may be sent to the bankers, Messrs. Barclay and Co., Ltd., 54 Lombard Street, E.C., or to the Treasurer (Sir G. Wyatt Truscott, Bart.), at the Hospital, Victoria Park, E.

**GREAT NORTHERN CENTRAL HOSPITAL.**—This institution serves extensive and populous districts in the north of London, and has treated a large number of sick and wounded soldiers. The endowment is very small, and help is always needed towards ever-growing maintenance expenses. Secretary, Mr. G. G. Panter.

**GUY'S HOSPITAL.**—With a total annual expenditure of between £80,000 and £90,000 a year, this great hospital is always in need of a share in public benefactions. The institution keeps itself well abreast of the times both in respect of the maintenance of its building and equipment, and as regards the excellence of its treatment of a large number of patients. Mr. Cosmo Bonsor is the Treasurer.

**KING'S COLLEGE HOSPITAL, DENMARK HILL, S.E.**—An appeal is being made to raise £100,000 to pay off the debt on the new buildings and to provide for current expenses. Up to the present only £16,000 has been received, and a further £4,000 is urgently required before the end of the year to enable the hospital to meet its immediate obligations. The Secretary is Captain H. S. Tunnard.

**LONDON HOSPITAL, WHITECHAPEL, E.**—The needs of this institution are very much the same as they were last year, and very much the same as the needs now felt by most hospitals. Donations and subscriptions are almost as high as they have ever been, but the chief difficulty is the extraordinary rise in expenditure, due to the increased prices, and it would seem that the general increases in price of commodities touches chiefly those with which the hospital has to deal. It is more expensive to provide hot water and to warm the wards because of the price of coal; it is more expensive to feed the patients because milk and bread and fish and meat and vegetables have all become dearer. A new item of expenditure in hospital accounts is that which has to be paid in the way of salaries, and is directly due to the war, which has taken away much unpaid service. The hospital also has to meet expenditure in that it provides certain payments to wives and families of those of its staff who have been called to the Front. These are some of the reasons why the hospitals need very warm support at this time. House Governor, Mr. E. W. Morris.

**LONDON HOMŒOPATHIC HOSPITAL.**—The board of management make a special appeal for aid in making good the large deficits incurred during the past few years. Seventy-five beds have been placed at the disposal of the Admiralty for sick and wounded sailors, of whom a large number have been treated. The receipt of contributions, addressed to the Earl of Donoughmore, the Treasurer, at the hospital, Great Ormond Street, W.C., will be gratefully acknowledged.

**METROPOLITAN HOSPITAL, KINGSLAND ROAD, N.**—In addition to the ordinary strenuous work of hospital service in a very poor and densely-populated district, accommodation has been provided for 296 sick and wounded soldiers. Immediate help is greatly needed. Mr. J. Courtney Buchanan is Secretary and House Governor.

**NATIONAL HOSPITAL FOR THE PARALYSED AND EPILEPTIC.**—The annual expenditure of this hospital has risen from £18,000 to £23,000, mainly in consequence of the increase in the prices of food and drugs. In the large out-patient department (50,000 attendances are made annually) a considerable quantity of bromides is prescribed, and the huge rise in price of this indispensable article has had a material effect on the accounts. Seventy soldiers suffering from shell shock, blindness, mutism, deafness, nerve injuries, and general nervous complaints are to be found in the wards. The beds have been increased to 170 to meet the extra call for in-patient treatment. An appeal for £10,000 is being issued by the board of management. The Secretary is Mr. Godfrey H. Hamilton, Queen Square, W.C.

**ROYAL FREE HOSPITAL, GRAY'S INN ROAD, W.C.**—The committee are making strenuous efforts to avoid getting into debt, and they urgently need support for maintenance and for extension of the maternity and infant welfare work of the hospital. Mr. Reginald R. Garratt, Secretary.

### FURTHER HOSPITAL APPEALS.

### Hospitals with 150 Beds and Upwards.

**ST. BARTHOLOMEW'S HOSPITAL, WEST SMITHFIELD, E.C.**—Owing to the great increase in the cost of provisions, fuel, and most other articles of daily consumption, and to other unavoidable charges directly attributable to the war, considerable additional support is essential. Mr. Thomas Hayes, Clerk.

**ST. GEORGE'S HOSPITAL, HYDE PARK CORNER, S.W.**—The secretary appeals for cigarettes, tobacco, games, etc., for the wounded and sick sailors and soldiers who are being treated at the hospital. Contributions also are urgently required for the general maintenance of the institution, in order that its work for the sick poor at home may be carried on as efficiently as heretofore.

**ST. MARY'S HOSPITAL, PADDINGTON, W.**—The regular sources of income yield about £18,000 only. A further sum of £17,000, therefore, is required every year. While this hospital is doing a great work for the war, its annual subscribers have fallen off through the war to the extent of £550 a year. Its war work consists not only in the devotion of one-third of its beds to the treatment of wounded soldiers from the Front, but in the manufacture and supply of anti-typhoid and other vaccines to all the Allied Armies in the field. No fewer than sixteen million doses have been supplied during 1916. To this work is due, in great measure, the striking rarity of typhoid fever in the Allied Forces. An interesting feature of the treatment of wounded soldiers in St. Mary's is an improvement in the dressing of wounds which originated in its wards. By the introduction of a thin sheet of celluloid between the raw wound and the dressing, the dreaded sticking of the latter, which renders its periodical removal so painful, has been prevented without detriment to the healing process. The gratitude of the soldier for this mitigation of his sufferings can be more easily imagined than described. The hospital has also taken a prominent part in the national campaign for the eradication of tuberculosis, and its new Tuberculosis Dispensary has been in full operation throughout the year. Secretary, Mr. Thomas Ryan.

**ST. THOMAS'S HOSPITAL.**—While this great hospital carries on its civil work and answers all the calls made upon it, as the 5th London General Hospital it plays a large part in dealing with the casualties of the war. A total income of not far short of £100,000 is required, and towards this large sum contributions are urgently needed. Mr. G. Q. Roberts is the Secretary.

**SEAMEN'S HOSPITAL, GREENWICH, S.E., AND ALBERT DOCKS.**—Over 350 men of the Royal Navy and Merchant Service are constantly under treatment, and the nation owes much to the courage, endurance, and skill of British seamen. Secretary, Mr. P. Michelli, C.M.G.

**UNIVERSITY COLLEGE HOSPITAL, GOWER STREET.**—This hospital, serving the northern districts of London, was rebuilt and enlarged 1897-1905. The hospital has its own Medical School and Private Nurses' Institution. There is an important Samaritan Fund. A special feature is the treatment by means of a complete set of medicinal baths. Funds for maintenance are much needed. The Secretary is Mr. J. G. T. Buckle.

**WESTMINSTER HOSPITAL, BROAD SANCTUARY, S.W.**—Since the beginning of the year 411 military patients have been received, and owing to the great advance in the price of supplies an appeal is made for generous support. Secretary, Mr. S. M. Quennell.

---

**ROYAL HOSPITAL FOR INCURABLES, PUTNEY HEATH.**—£35,000 is needed annually to provide a home for incurables above the pauper class, and for those having a home but no means of support, a pension of £20 a year. Mr. Charles Cutting, Secretary.

